# In-depth Genetic and Molecular Characterization of Unilateral Coexisting Adrenal Cortical Adenoma and Carcinoma in the Context of MEN1 Syndrome

**DOI:** 10.1007/s12022-026-09908-0

**Published:** 2026-03-10

**Authors:** Francesca Cioppi, Tommaso Orioli, Giulia Cantini, Tonino Ercolino, Federica Cioppi, Guillaume Assié, Anne Jouinot, Anna Aurora Dedonno, Raffaella Santi, Ronald R. de Krijger, Mario Maggi, Matteo Benelli, Letizia Canu, Gabriella Nesi, Michaela Luconi

**Affiliations:** 1 Department of Experimental and Clinical Medicine, University of Florence, Florence, 50139 Italy; 2 European Network for the Study of Adrenal Tumors (ENSAT) Centre of Excellence, University of Florence, Florence, 50139 Italy; 3 Department of Experimental and Clinical Biomedical Sciences, University of Florence, Florence, 50139 Italy; 4 Centro di Ricerca & Innovazione Sulle Patologie Surrenaliche, AOU Careggi, Florence, 50134 Italy; 5 Azienda Ospedaliero-Universitaria Careggi, (AOUC), Florence, 50139 Italy; 6 Université Paris Cité, CNRS, INSERM, Institut Cochin, Paris, F-75014 France; 7 Department of Health Sciences, University of Florence, Florence, 50139 Italy; 8 Princess Máxima Center for Paediatric Oncology, Utrecht, The Netherlands; 9 Department of Pathology, University Medical Center Utrecht, Utrecht, 3584 CX The Netherlands

**Keywords:** Whole Exome Sequencing, MEN1, Clonal evolution, ACC and ACA, Somatic mutations, Molecular profiling

## Abstract

Adrenal lesions often occur in patients with multiple endocrine neoplasia type 1 (MEN1), mostly adrenal cortical adenomas (ACAs), although the frequency of adrenal cortical carcinomas (ACCs) is higher than in the general population. The coexistence of benign and malignant masses has seldom been documented, leaving open the question of ACC progression from benign forms. We report a comprehensive genetic characterization of three adrenal cortical tumor samples obtained from a familial MEN1 patient, operated for the rapid progression of an initially stable nonfunctional adrenal incidentaloma. Histologically, the tumor consisted of a small ACA contiguous to a large ACC, which subsequently relapsed. Exome sequencing of ACC, ACA and recurrence evidenced a *MEN1* loss of heterozygosity (LOH) in ACC but not in ACA, where, however, a second hit driven by alternative mechanisms could not be excluded. The majority of the ACA variants were found to co-occurred in ACC (*n* = 36/42) and were benign, except for two of unknown significance in *KANK1* and *REN* genes, described as associated with renal cancer. Among variants shared between ACC and its recurrence (*n* = 69), 11 were Tier III, while 2 affecting *TP53* and *NF1* genes were pathogenic. Bioinformatic clonal evolution analysis identified one clone − characterized by *TP53* and *NF1* mutations − absent in ACA but present in ACC and recurrence, as well as 2 clones shared between ACA and ACC but lost in the recurrence. In conclusion, comparative Whole Exome Sequencing (WES) analysis of three adrenal tumors in a MEN1 patient suggests a possible relationship between malignant and benign lesions occurring in MEN1 patients, without, however, demonstrating any causal adenoma-to-carcinoma progression driven by MEN1 LOH. Overall, these data further suggest an increased risk of MEN1 patients to develop adrenocortical malignancy.

**Supplementary Information:**

The online version contains supplementary material available at 10.1007/s12022-026-09908-0.

## Introduction

Multiple endocrine neoplasia type 1 (MEN1) is a highly penetrant autosomal dominant disorder caused by germline pathogenic variants in the *MEN1* gene (11q13.1), and characterized by the occurrence of tumors in two or more endocrine glands, particularly the parathyroids, pancreas and anterior pituitary gland [1]. Although not usually the first manifestation, adrenal lesions are common in MEN1, occurring in 20.4–55% cases in clinical series [2–4], with an even higher prevalence (73%) reported in endoscopic ultrasound series [5]. This is a dramatic increase compared to the general population, where the frequency of incidentalomas is approximately 3% [6]. Moreover, in MEN1, the ACC prevalence of 4.4−5.4% [2–4] is higher than the 0.4−4% reported in the general population [6], confirming the aggressive clinical behavior of adrenal tumors [7–10].

In the Gatta-Cherfili’s study cohort of 146 MEN1 patients with adrenal involvement, 10 ACCs (9 classified as stage I or II, and 1 as stage IV) were histologically diagnosed in 8 of the 45 patients operated on due to an increase in the size of the mass detected by computed tomography (CT) [2]. Similar results were obtained in a cohort of 89 MEN1 patients with an instrumental diagnosis of adrenal incidentaloma, where 2 out of 12 surgically removed masses showed morphological features of ACC (16.7%) [4].

In MEN1 patients, adrenal incidentalomas are most commonly discovered during routine imaging and are often non-secreting, making active surveillance a clinical challenge due to the limited availability of reliable predictors of tumor behavior and progression. In this context, determining whether a malignant adrenal tumor arises from a pre-existing benign lesion is particularly relevant for patient management. The possibility of malignant transformation versus an independent multifocal origin (collision hypothesis) remains a matter of debate in the study of adrenal cortical tumors [11, 12].

Whole Exome Sequencing (WES) is a valuable tool for understanding cancer progression from a premalignant lesion and for elucidating the relationships between concomitant tumors. It enables the detection of low-frequency variants, which can be critical for investigating the clonal origin of tumors [13, 14].

Here, we report a patient with familial MEN1, who underwent surgery for the rapid enlargement of a previously stable, nonfunctional adrenal incidentaloma. Pathology revealed an extended component with malignant features indicative of ACC contiguous to a small peripheral mass exhibiting morphological characteristics of an adrenal cortical adenoma (ACA). The patient experienced a relapse in the diaphragmatic bed 2 years later. Tissue availability from the two coexistent tumors and the recurrence allowed a unique comparative molecular characterization by deep genetic profiling of the three lesions to assess their lineage relationships in the context of MEN1.

## Methods

### Ethics

This study was designed and conducted in accordance with the Declaration of Helsinki. The study was approved by the local Ethical Committee (Prot. 2011/0020149), and the recruited patient gave informed consent to the study.

### Pathological Analysis

Histological diagnosis was carried out by two independent reference pathologists on the tumor tissues removed at surgery (ACC, ACA, and recurrence) according to established diagnostic criteria [15]; see Supplementary File 1.

### Transcriptome Signature

Transcriptome analysis was performed at Institut Cochin, as previously described [16]; see Supplementary File 1.

### Molecular Genetics

Blood DNA and DNA from the frozen recurrence specimen were extracted using DNeasy Blood & Tissue Kit (Qiagen, Germany), according to the manufacturer’s instructions. Tumor DNA was extracted from the FFPE ACA and ACC samples, according to the manufacturer’s instructions. DNA quality and quantity were measured by the Qubit BR assay (ThermoFisher Scientific, USA).

*Sanger sequencing* was performed to analyze all coding exons along with exon-intron boundaries of the *MEN1* gene and to validate the selected variants by Next Generation Sequencing (NGS) analysis.

*Targeted NGS panel*: the ACC sample was analyzed using a previously validated targeted panel of 10 driver genes in ACC [17].

*WES and bioinformatics analysis*: patient DNA from different tissues (blood, ACA, ACC, and recurrence) underwent WES. Blood DNA was used to exclude germline variants. Exome sequencing was done by Cogentech SRL, using the Twist Exome 2.0 for WES library preparation, Illumina NovaSeq 6000 as a sequencing platform, and requesting a minimum coverage of 100X for blood and 200X for the other samples. Sequences were analysed by the Sarek workflow from nf-core [18]. Germline variants in the blood sample were detected by HaplotypeCaller-GATK version 4.5.0.0 [19]. For somatic alterations, Single Nucleotide Variants (SNVs) and small Insertion/Deletions were identified using Mutect2-GATK version 2.2 [19], Strelka version 2.9.10 [20] and VarScan version 2.3.9 [21], and ASCAT version 2.5.3 for somatic copy-number alteration (CNA) [22]. Cancer Cell Fraction (CCF) was computed as previously reported [23], using tumor purity and CNA from ASCAT.

Somatic variants with allele depth (AD) ≥ 20 and VAF ≥ 5% recomputed by Samtools version 1.20 [24] were considered for downstream analysis. To evaluate potential misclassification of subclonal variants using a 5% VAF threshold, we assessed the reliability of this threshold by comparing VAF with CCF (Fig. S1). Variants were classified according to the 5% cutoff for both measures. Overall, agreement was 91%, with 10 variants (9%) showing discordance (CCF ≥ 5% but VAF < 5%), with borderline CCF values (mean = 0.08, range = 0.05–0.10). Consequently, we used VAF = 5%, given its widespread application. Variants were classified as ACA exclusive (private to ACA), ACA + ACC (shared between ACA and ACC) and ACC+recurrence (shared between ACC and recurrence). To reduce the risk of false positives, a minimum read depth of 250 for the FFPE ACA and ACC samples [25] and 100 for the frozen recurrence was required. Concerning indels, only those called by two or more tools were considered. After annotating variants with VEP version 111, only coding SNVs and indels were considered for downstream analyses and variants were classified according to four tier categories of clinical significance in cancer diagnosis, prognosis and/or therapeutics (Fig. 3a & S3). Clonal analysis was done with PyClone (v.0.13.1) [26], using the final set of mutations and CNA and purity inferred by ASCAT.

## Results

### Clinical Characterization

We report the case of a woman affected by familial MEN1 syndrome with primary hyperparathyroidism, prolactin-secreting pituitary microadenoma, and multiple pancreatic neuroendocrine tumors (maximum diameter 11 mm). After 9 years, a left adrenal incidentaloma was reported (maximum diameter 12 mm, < 10HU) at CT scan. The patient was monitored according to the Guidelines for MEN1 management [9, 27]. The lesion increased in size to 18 mm over the next 5 years, and reached 50 mm after 5 additional years. An R0 laparoscopic adrenalectomy was carried out, disclosing an ACC (ENSAT stage II, Ki67 10%, Weiss score 6) coexistent with a small peripheral ACA. Two years later, the patient underwent laparoscopic surgery for ACC recurrence in the adrenal bed. Following surgery, mitotane therapy was started, achieving levels ≥ 14 mg/l. Due to a further local recurrence after 4 months, first-line chemotherapy with etoposide, doxorubicin and cisplatin (EDP) was initiated. Chemotherapy was interrupted after the third cycle, while mitotane was continued. After 10 months, the disease had progressed with lymph node and bone metastases, and the patient was administered six cycles of second-line therapy with cisplatin plus nivolumab in addition to radiotherapy to alleviate bone pain. As disease continued to progress, a third-line temozolomide regimen was proposed followed by a fourth-line capecitabine regimen. The patient died 3 years following surgical resection of the recurrent tumor.

### Differential Classification of the Three Tumors According to the Histological Oncomarkers

**Fig. 1:**
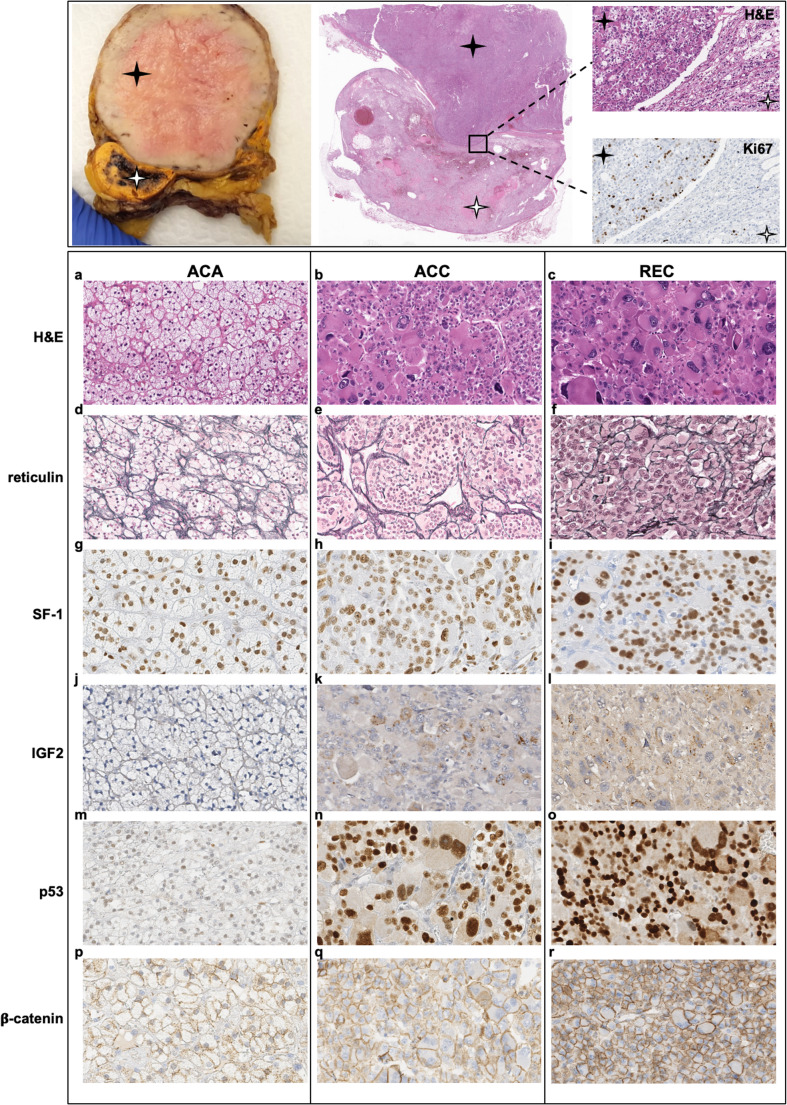
Pathological features of ACA, ACC and ACC recurrence: Upper panel. Gross morphology of the resected adrenal mass (left) showing a large ACC (black star) adjacent to a small ACA (white star); H&E staining of the two nodules (center) with magnification revealing variation of Ki67 labeling indices in ACC and ACA (right). Lower panel: comparative histochemical and immunohistochemical characterization of ACA (a, d,g, j,m, p), ACC (b, e,h, k,n, q) and ACC recurrence (c, f,i, l,o, r). Panels** a-c**: Profound cytological and architectural atypia in ACC and ACC recurrence (REC) compared to ACC histology, resembling the normal adrenal *zona fasciculata*. Panels **d-f**: Preserved (ACA) vs. disrupted (ACC and ACC recurrence) reticulin framework. Panels** g-i**: Diffuse nuclear SF1 immunoreactivity in the three lesions. Panels** j-l**: IGF2 dot-like paranuclear staining was evident in the two malignancies but negative in ACA. Panels **m-o**: Wild-type p53 reactivity in ACA and high p53 expression in ACC and recurrence. Panels **p-r**: β-catenin expression limited to the cell membrane in the three lesions

Macroscopic and microscopic features of the adrenal tumor and of the recurrent lesion are shown in Fig. 1.

Grossly, the adrenal lesion was 65 × 55 × 50 mm in size and weighed 120 g. On cut section, it consisted of two distinct coexisting nodules: the larger (maximum diameter 50 mm) was light tan in color, with a central area of necrosis, while the smaller (maximum diameter 15 mm) was golden yellow with no necrotic or hemorrhagic changes. Compressed adrenal cortical tissue was noted at the periphery (Fig. 1 upper panel).

On microscopic examination, the larger nodule (Fig. 1) was composed of pleomorphic eosinophilic cells with high nuclear grade, brisk mitotic activity (12 mitoses/10mm^2^), and a diffuse growth pattern. Necrotic areas and vascular invasion were seen. The smaller nodule (Fig. 1) was constituted by clear cells in small nests and chords, resembling the fasciculata zone of the adrenal cortex. Well-consolidated histopathological biomarkers were assessed to further validate the differential diagnosis of the two masses [28]. The reticulin framework appeared intact in the smaller nodule, but disrupted in the larger, in line with its previously demonstrated diagnostic relevance [29] (Fig. 1). Immunohistochemistry revealed SF1 positivity in both lesions as well as in the recurrence, establishing their adrenocortical origin [28] and proliferative characteristics, given that SF1 dosage correlates with increased proliferation in human adrenocortical cells and promotes adrenal tumor formation in mice [30]. The larger nodule showed diffuse strong immunoreactivity for p53, dot-like paranuclear staining for the paracrine factor Insulin-like Growth Factor 2 (IGF2) (Fig. 1), and a Ki67 labeling index of 10% (Fig. 1 inset), features widely used to differentiate benign from malignant lesions of the adrenal cortex [31–33]. In contrast, the smaller lesion displayed a Ki67 labeling index of 2%, no mitotic figures, a wild-type p53 staining pattern, and no IGF2 expression (Fig. 1). Membranous β-catenin expression was seen in both lesions, with increased intensity in the malignant form (Fig. 1). Based on the described biomarkers, a histological diagnosis of ACA and ACC was established for the smaller and larger lesions, respectively. Tumor recurrence exhibited morphological and phenotypic features consistent with prior ACC (Fig. 1).

### Differential Gene Expression in ACC and ACA

To assess if the functional significance of the *TP53* variant found is associated with an upregulation of protein expression, described as “gain of function” [34], we explored protein expression by immunohistochemistry. The strong nuclear p53 staining observed in ACC and the recurrence, together with the very low-level staining seen in ACA, supported an altered TP53 pattern in the malignant lesions and a wild-type pattern in ACA (Fig. 1m-o). Increased p53-target β-catenin was associated with ACC, though positivity was limited to the cell membrane (Fig. 1p-r).

**Fig. 2:**
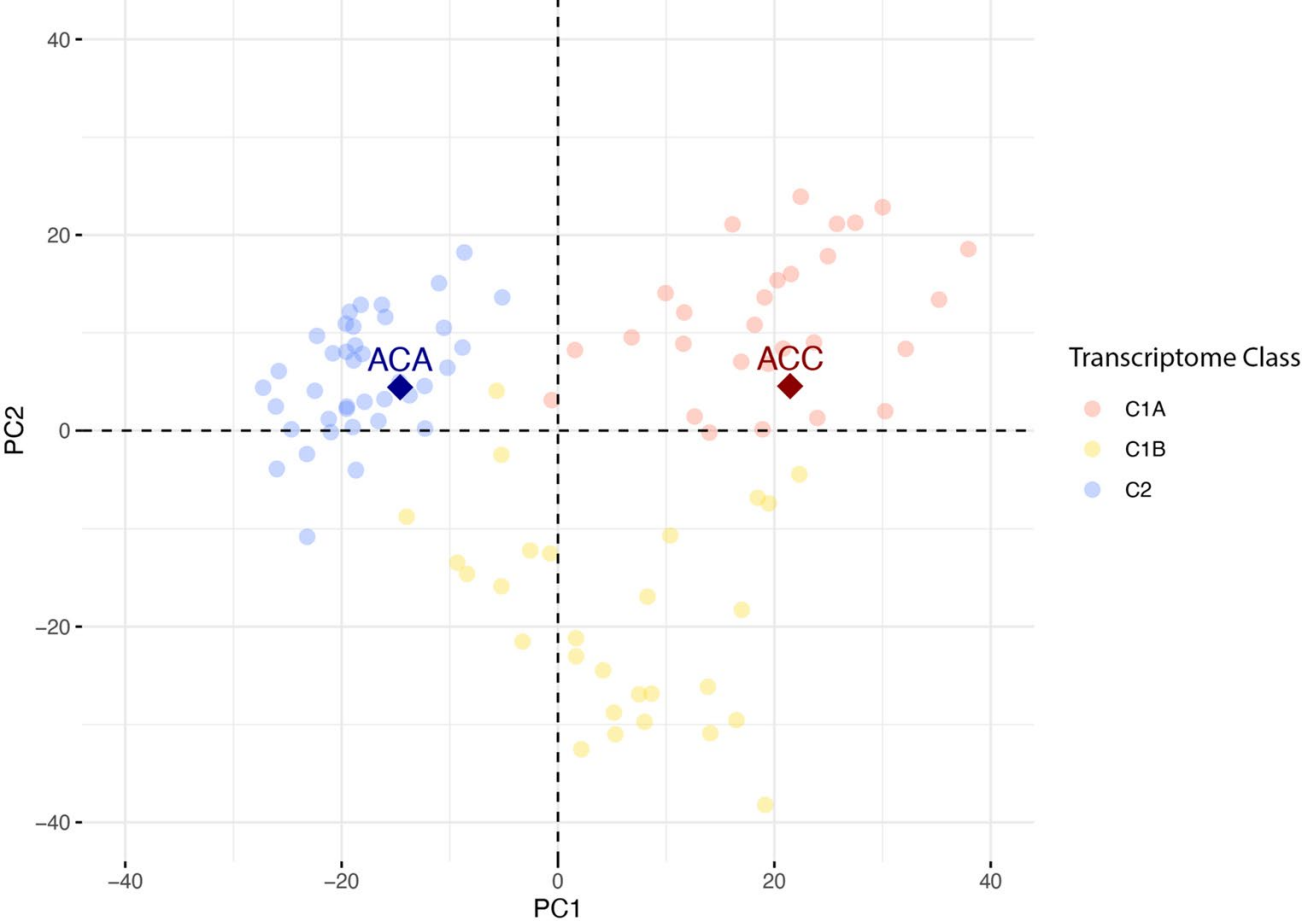
FFPE transcriptome classification of adrenal tumor samples. Patient’s samples (⬪) were projected on the two first axes (PC1, PC2) of the principal component analysis (PCA) built in a reference cohort of 95 patients [16]. Samples from this reference cohort are presented as faint circles colored by transcriptome class: blue for adrenal cortical adenomas “C2”, red for adrenal cortical carcinoma of poor prognosis “C1A” and yellow for adrenal cortical carcinoma of better prognosis “C1B”

Application of 3’-end RNA sequencing transcriptome technology to FFPE of the primary adrenal tumor samples correctly classified ACC (C1A, Fig. 2) and ACA (C2, Fig. 2) on the basis of a predictive transcriptome model previously developed [16], with a score of 93.3% and 99.1%, respectively. According to the model, ACC transcriptome profile was associated with a high risk of recurrence (Fig. 2).

### Loss of Function Variant in MEN1

Sanger sequencing identified a heterozygous germline frameshift mutation in the exon 8 of *MEN1* (NM_001370259.2): c.1154del, p.(Ala385GlyfsTer60) (Fig. S2). This variant was novel and was classified as pathogenic, according to the recommendation of the American College of Medical Genetics and Genomics (ACMG) [35]. *MEN1* gene sequencing in the different tumor samples suggested a loss of heterozygosity (LOH) both in ACC and recurrence but not in ACA (Fig.S2).

### Targeted NGS Analysis

The targeted NGS panel we recently published [17] identified two somatic variants with strong clinical significance (Tier I) and high frequency (≥ 70%), Table 1).

**Table 1:** List of the selected gene variants identified by targeted NGS in the ACC sample. Transcripts, changes on cDNA and protein, variant type, tier classification [36], ACMG classification, COSMIC identifier (ID) and Variant Allele Frequency (VAF) are reported for both variants. n.a.: not available; p.? indicates that the variant occurs in a noncoding sequence of the gene

Gene	Transcript	cDNA	Protein	Variant type	Tier	ACMG	Legacy ID (COSMIC)	VAF
***NF1***	NM_000267	c.4270-1G > C	p.?	splicing	I	Pathogenic	n.a.	70.85%
***TP53***	NM_000546	c.376T > G	p.Tyr126Asp	missense	I	Pathogenic	COSM6024609	72.45%

### Exome Analysis of ACA, ACC and Recurrence

In order to explore the somatic mutational profiles of the three tumors and evaluate possible correlations among the three tumor conditions, we performed parallel WES analysis of patient DNA from blood and tumor tissues, which resulted in a list of somatic tumor-only variants, some in common and some specific of each tumor conditions.

### Molecular Characterization of the Tumors

**Fig. 3:**
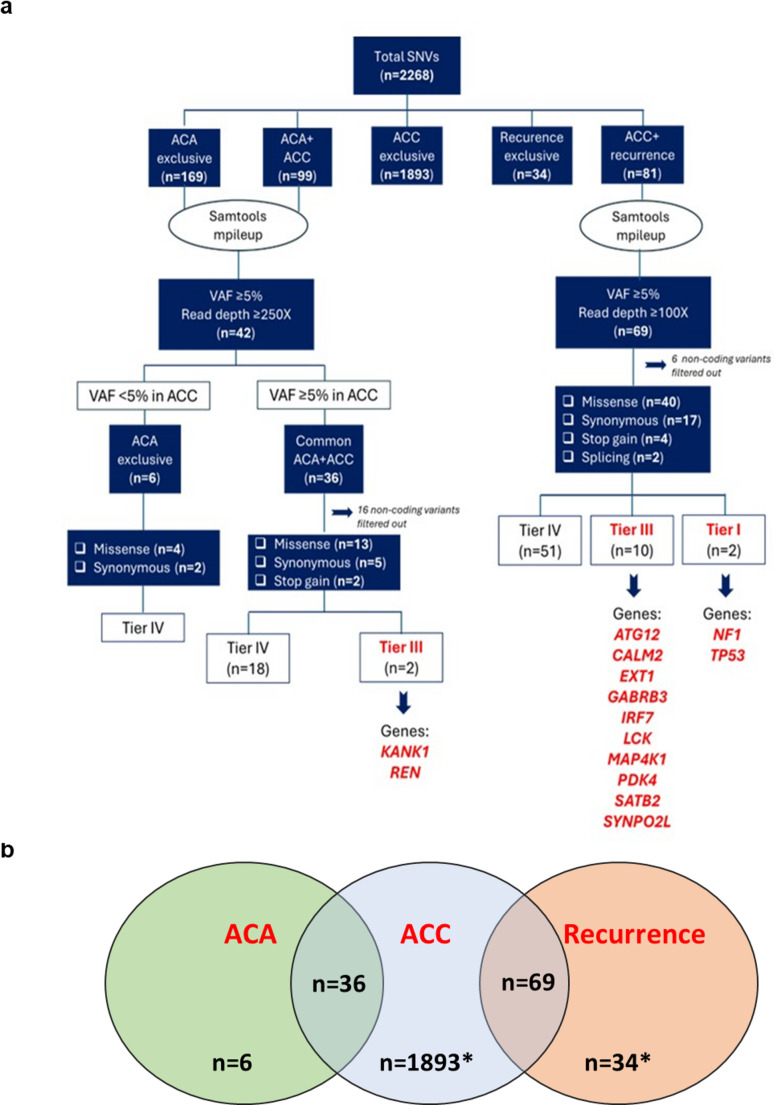
SNVs filtering with final retained variants in relevant genes. Panel **a**: Flowchart illustrating the number of total and prioritized variants in the different tumor samples after the variant filtering approach. Red color indicates affected genes by variants of unknown significance or pathogenic. SNVs: Single Nucleotide Variants; Tier IV: variants classified as likely benign/benign; Tier III: variants of unknown clinical significance; Tier I: variants with strong clinical significance; VAF: Variant Allele Frequency. Panel **b**: Venn diagram showing the final number of exclusive or shared SNVs in different tumor samples. *indicates variants not checked by SAMtools pileup

We found 2268 SNVs (Fig. 3) and 1594 indels (Fig. S3). Since all indels were classified as benign/likely benign, and only 2/9 were with unknown significance (Fig. S3), we focused on SNVs. Neither SNVs nor indels co-occurred in ACA and recurrence.

Applying thresholds of VAF ≥ 5% and read depth ≥ 250x in FFPE-ACA tissue, 42 variants remained. Allele frequency in the ACC sample was thoroughly established by SAMtools pileup, obtaining a total of 6 ACA-exclusive and 36 ACA/ACC-common variants. Interestingly, all filtered variants were rare. All ACA-exclusive SNVs, consisting of 4 missense and 2 synonymous variants, were classified as Tier IV (benign or likely benign). ACA/ACC-common SNVs in coding regions included 13 missense, 5 synonymous and 2 stop gain, two classified as Tier III (Fig. 3a). A stop gain variant in *KANK1* gene (NM_015158.5: c.3342T > A p.(Cys1114Ter) was present in both ACA and ACC (Table 2).

**Table 2:** List of prioritized somatic gene Variants, i.e. Tier III/I, with the corresponding chromosomal location and sequence variant nomenclature according to HGVS. Variant allele frequency in ACA, ACC, recurrence, variant classification according to the ACMG and Tier/AMP guidelines, variant identification in cBioPortal and gene-associated cancer are also reported. LP: likely pathogenic; P: pathogenic; VAF: VriAnt allele Frequency; VUS: Variant of Unknown Significance

Gene	Locus	HGVS	VAF in ACA (%)	VAF in ACC (%)	VAF in recurrence (%)	ACMG	Tier	cBIO Portal	Gene-associated cancer*
*ATG12*	5q22.3	NM_004707.4:c.416G > A p.(Trp139Ter)	0	36.1	29.2	VUS	III	Pancreatic cancer	*Liver*,* ovary*,* kidney*,* thyroid*
*CALM2*	2p21	NM_001743.6:c.73G > T p.(Asp25Tyr)	0	60	55,1	VUS	III	n.a.	*breast*,* colorectal*,* liver*,* pancreas*,* kidney*
*EXT1*	8q24.11	NM_000127.3:c.1031 C > G p.(Ser344Cys)	0	35.6	30.8	P	IV	n.a.	*Lung*
*GABRB3*	15q12	NM_000810.4:c.409 A > G p.(Asn137Asp)	0	38	36.8	LP	III	n.a.	Prostate adenocarcinoma
*IRF7*	11p15.5	NM_001572.5:c.1252 C > T p.(Arg418Trp)	0	68.3	54.9	VUS	III	n.a.	*Liver*,* urothelial*
*KANK1*	9p24.3	NM_015158.5:c.3342T > A p.(Cys1114Ter)	5	7.54	0	LP	III	n.a.	*Kidney*
*LCK*	1p35.2	NM_005356.5:c.373G > A p.(Glu125Lys)	0	34.5	37.5	VUS	III	Glioma Melanoma	T-cell acute lymphoblastic leukemia
*MAP4K1*	19q13.2	NM_001042600.3:c.865G > C p.(Asp289His)	0,7	43.1	33	VUS	III	n.a.	Lymphoma
*NF1*	17q11.2	NM_001042492.3:c.4333-1G > C p.?	0	65.9	56.9	P	**I**	Bladder cancer	Breast
*PDK4*	7q21.3	NM_002612.4:c.625G > C p.(Glu209Gln)	0	38.7	31.7	VUS	III	n.a.	Kidney, Thyroid
*REN*	1q32.1	NM_000537.4:c.995 C > A p.(Pro332His)	8.61	6.85	0	VUS	III	n.a.	Kidney
*SATB2*	2q33.1	NM_001172509.2:c.1547G > A p.(Trp516Ter)	0	69.4	53.6	LP	III	n.a.	Bowel
*SYNPO2L*	10q22.2	NM_001114133.3:c.235 C > T p.(Gln79Ter)	0	34.9	37.8	LP	III	n.a.	Breast, Head and Neck and Prostate.
*TP53*	17p13.1	NM_000546.6:c.376T > G p.(Tyr126Asp)	0	67.3	47.4	P	**I**	18 cancer types#	Breast, colorectal, lung, sarcoma, prostate, adrenal cortical, glioma, Spitzoid tumor, multiple other tumor types

*based on cancer specificity/prognostic for cancers (italic form) according to Human Protein Atlas. #esophagogastric cancer, colorectal cancer, glioma, non-small cell lung cancer, breast cancer, hepatobiliary cancer, pancreatic cancer, soft tissue sarcoma, renal cell carcinoma, prostate cancer, myelodysplastic syndrome, melanoma, liver cancer, head and neck cancer, gastrointestinal neuroendocrine tumor, endometrial cancer, mature B-cell neoplasms, esophageal-stomach cancer

Regarding filtering of co-occurring SNVs in ACC and recurrence, a threshold of ≥ 100x read depth was applied to all detected variants with a VAF ≥ 5%, obtaining 69 SNVs. After the exclusion of non-coding ones, 40 missense, 17 synonymous, 4 stop-gain and 2 splicing variants were selected. Among them, 10 variants were classified as Tier III, since all affected genes were found to be associated with cancers (Table 2). The only two variants classified as Tier I, and therefore considered as pathogenic, remained those associated with *NF1* and *TP53* genes.

To validate the relevance of the mutated genes found in ACA, ACC and recurrence, along with *MEN1*, we assessed their frequency in a large independent ACC cohort, querying the TCGA-ACC Firehose Legacy [37]. The OncoPlot (Fig. S4) showed the frequency in the TCGA database of each mutated gene, classified according to the presence in ACA, ACC and recurrence, also reporting their association with *TP53*,* NF1* and *MEN1* somatic mutations, as found in TCGA. The top 12 most mutated genes in TCGA-ACC samples were found to be wild-type in ACA, suggesting that these genes are not shared with the benign tumoral forms. Among the genes harboring mutations shared between ACA and ACC, *FAM47E*,* SLC26A2*, and *WDR7* displayed a 2% mutation frequency in the TCGA cohort, whereas somatic mutations in *KANK1* gene reached only 1% (Fig. S4).

**Fig. 4:**
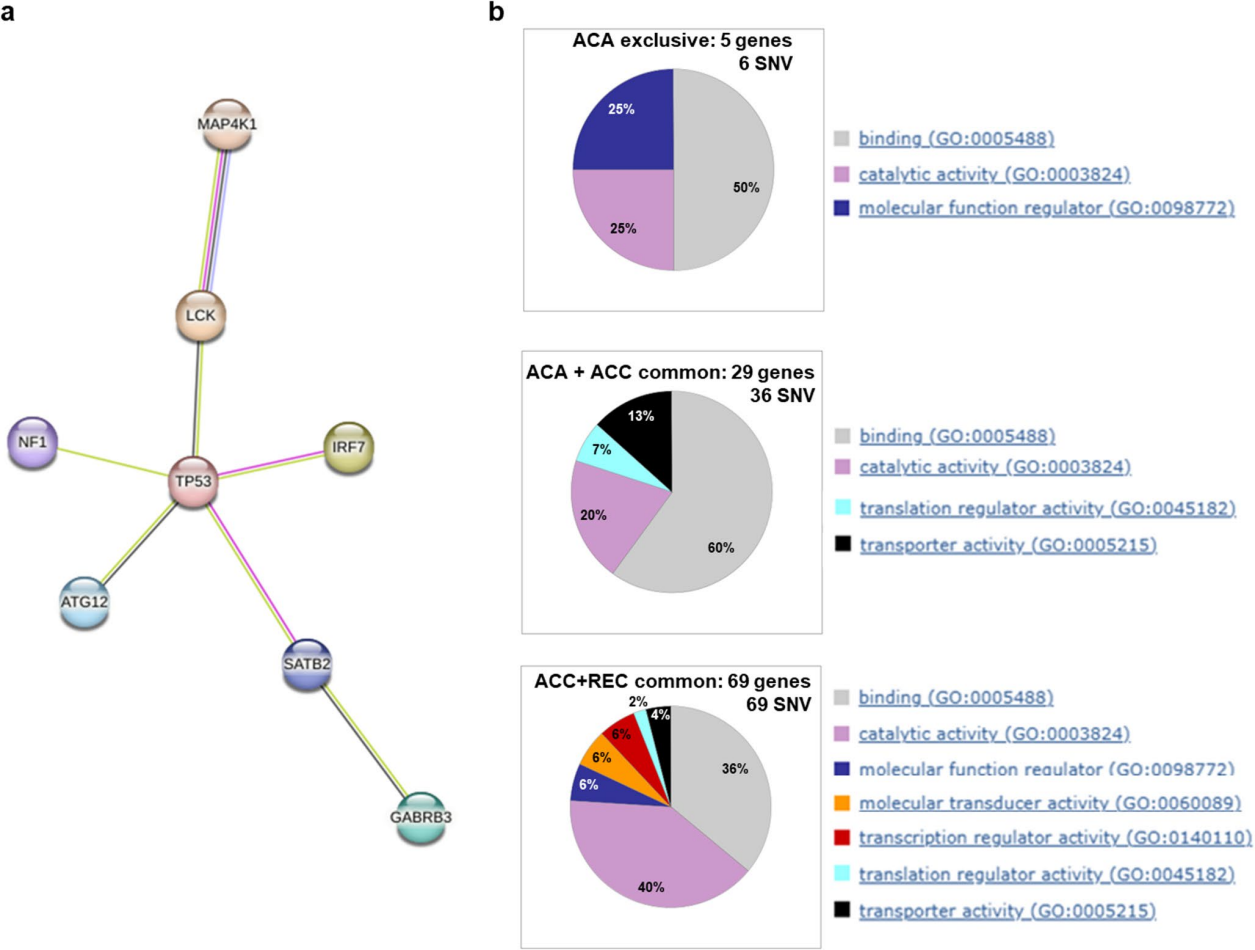
Clustering by function and interaction between proteins encoded by genes found mutated in the three tumor conditions. Panel a. STRING analysis of interactive pathways shows that TP53 represents the central node of the protein interactive network involving 8 out of 12 genes identified to bear SNV in the malignant trajectory exclusively shared by ACC and recurrence. Panel** b**. Gene ontology enrichment analysis performed through PanGo Human Functionome (https://functionome.geneontology.org). GO-PANTHER software shows the distribution of the molecular function protein clusters corresponding to the mutated genes in the three tumor conditions: ACA exclusive genes, ACA + ACC common genes, and ACC+recurrence common genes. Molecular functional classes according to Go-Panther ontology are indicated to the right, and their percentage distribution inside the pie graphs

Notably, 8/12 “driver” genes with malignant potential, exclusively present in ACC and recurrence, showed predicted protein interactions centered in TP53, according to the STRING analysis of protein-protein interaction networks (Fig. 4a). Figure 4b reports gene ontology enrichment analysis, which clusters mutated genes according to their molecular functions in the three conditions (ACA exclusive, ACA + ACC common, or ACC + REC common). Cluster distribution showed that all functional clusters common to ACA and ACC were also maintained in the recurrence, as well as three additional classes, corresponding to transcriptional regulators, molecular function regulators and molecular transducers emerged as being shared by ACC and recurrence, but not by ACA.

**Fig. 5:**
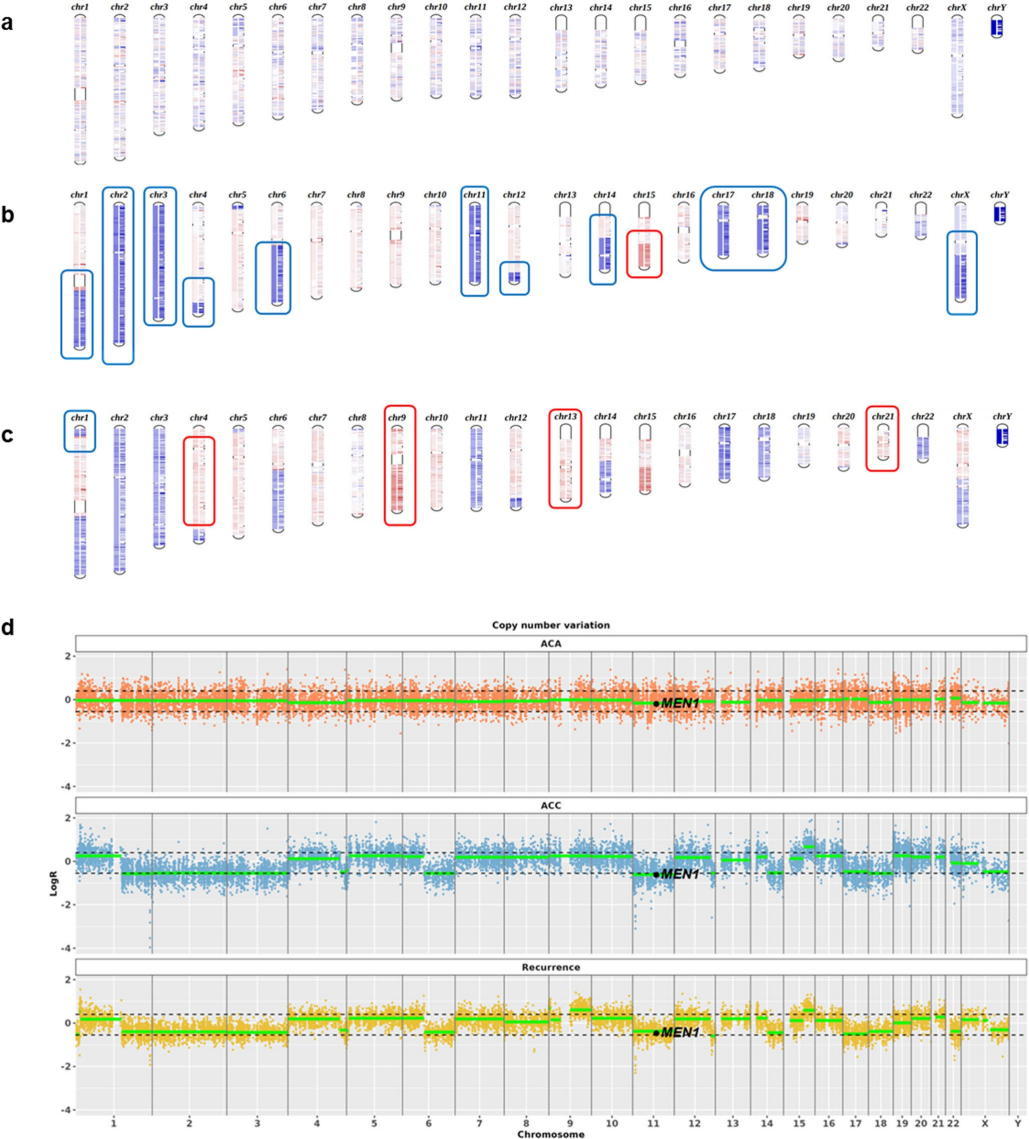
CNA in ACA, ACC and recurrence. Ideogram illustrates the overall landscape of gains (red color) and losses (blue color) in ACA (panel a), ACC (panel b) and recurrence (panel c) across each chromosome. Rectangles highlight copy number changes compared to germline (panel **b**) and to ACC condition (panel c). Panel **d**. CNA profile with ASCAT data in the three conditions is shown. ACA (upper panel) retained heterozygosity for the MEN1 locus (11q13), whereas LOH at this locus is evident in both ACC (middle panel) and recurrence (lower panel)

To integrate the information derived from SNV and indel analysis for tracing common or unique somatic alterations of ACC, ACA and recurrence, we also analyzed duplications and deletions of genes across the chromosomes. Copy number alteration (CNA) analysis of the 3 tumor samples (Fig. 5) revealed no alteration in ACA (Fig. 5a), while ACC (Fig. 5b) and recurrence (Fig. 5c) showed pervasive arm-level copy number changes.

CNA patterns were concordant between ACC and the recurrent tumor, with both exhibiting *MEN1* LOH. Contrariwise, no alterations at the *MEN1* locus (11q13) were detectable in ACA (Fig. 5d), as indicated by WES analysis and Sanger sequencing.

**Fig. 6:**
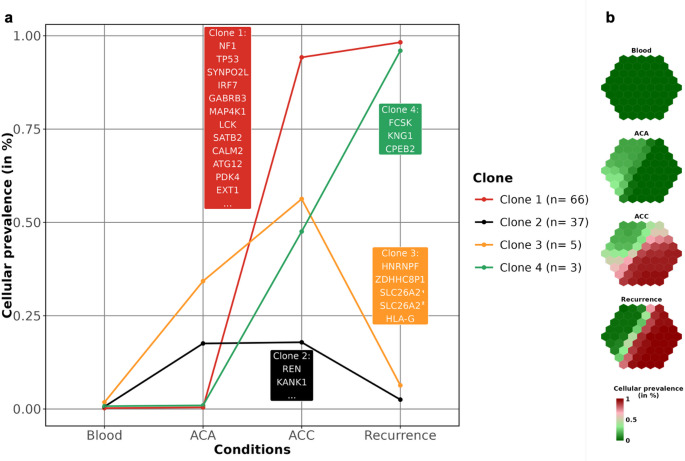
Cellular prevalence of clonal populations in blood, ACA, ACC and recurrence shown as clusters and as set of mutations. Panel** a**. Since PyClone creates clusters of mutations that have a different evolution in different tissues, each clone has a different cellular abundance in different conditions, suggesting a different distribution in the four conditions. Clones are numbered by mutation counts. In legend, the number of SNVs for each clone is reported. Some of the genes in clone 1 and 2, which are the most interesting as bearing the highest number of mutations, are indicated in the boxes; clone 3 bears 2 different mutations on the same gene. *Clone 3 contains two mutations in *SLC26A2*. Panel** b**. Cellular prevalence of each clone is summarized in a single hexagon where each cell represents a set of mutations and the relative abundance in the four conditions. Hexagon created with supraHex R package (v.1.10.1) [38]

The curated set of SNVs (*n* = 111) were used to infer clonal populations and potential relationships across samples (Supplementary File 2.xlsx). PyClone analysis identified four distinct clones (Fig. 6a). Two clones (clones 1 and 4) were shared between ACC and the recurrence. Clone 1, which included all Tier III-classified variants along with pathogenic *TP53* and *NF1* mutations, was the most prevalent, suggesting a relevant role in disease progression. Clone 4 displayed increased clonality in the recurrence compared to ACC. Two additional clones, comprising 37 Tier IV variants (clone 3) and two Tier III variants in *KANK1* and *REN* (clone 2), were shared between ACA and ACC but were not detected in the recurrence, which may be consistent with a divergent evolutionary pattern compared to clones 1 and 4. No clones were specific to ACA. These results were supported by CCF analysis, showing comparable trends across the different clones (Fig. S5).

## Discussion

In this study, the unique simultaneous presentation of ipsilateral ACA and ACC, along with the availability of a subsequent recurrence, enabled the morphological and molecular comparison of the three tumors to investigate: (i) the progression versus collision hypothesis of ACA and ACC in the context of MEN1 syndrome; (ii) the identification of driver clones with malignant potential.

The coexistence of ACC and ACA, so far only rarely reported—with limited genetic characterization [12, 39, 40] and all but one outside the context of MEN1—suggests tumor collision as the most likely scenario. A recent paper summarized sporadic cases of malignant transformation of adrenal incidentalomas, likely ACAs, into ACCs [41]. However, in these cases, the diagnosis of benign lesions was based exclusively on imaging, without histopathological confirmation. The strongest evidence against a stepwise progression from ACA to carcinoma lies in the striking discrepancy between the high prevalence of adenomas and the rarity of ACCs. Nonetheless, in MEN1, the frequency of incidentalomas increases together with that of carcinomas, implying a greater risk of malignancy in MEN1.

In our case, WES analysis revealed significant arm-level copy number changes in ACC and its recurrence, in line with previous reports [37, 42, 43], with largely overlapping CNA patterns between the two malignant lesions. In contrast, ACA exhibited a neutral copy-number profile, suggesting that CNAs may contribute to adrenocortical malignancy.

Concerning SNVs and indels, we applied stringent filtering criteria to identify a reliable set of somatic variants, which were classified into four Tier categories based on their clinical impact. SNVs shared between ACC and the recurrence, but not detected in ACA, are in keeping with a clonal relationship between the two malignant lesions and support their interpretation as a true relapse. Samtools pileup and filtering by ≥ 2 somatic variant calling tools applied to SNVs and indels yielded a reduced, high-confidence variant set, comprising six SNVs exclusive to ACA, 36 shared between ACA and ACC, and 69 shared between ACC and the recurrence. No variants were identified in genes previously reported to predispose to ACA [44], and all ACA-exclusive SNVs were classified as benign. Among ACA-ACC shared mutations, two were classified as Tier III and affected *KANK1* and *REN* genes. *KANK1* is a candidate tumor suppressor gene for renal cell carcinoma [45, 46] and has been reported as mutated in the TCGA-ACC cohort, albeit at a low frequency of approximately 1%. A potential involvement of *KANK1* haploinsufficiency in centrosome aberrations has been documented in tumorigenesis [47]. The relatively high number of SNVs shared between ACA and ACC, compared with the limited number of ACA-exclusive variants, is compatible with a possible relationship between the two lesions.

Of the 69 SNVs shared between ACC and the recurrence, 10 variants were of unknown significance (Tier III) and 2 of strong clinical significance (Tier I), including those in the *TP53* and *NF1* genes, which are frequently mutated in ACC [17]. Immunohistochemistry demonstrated strong p53 overexpression confined to ACC and retained in the recurrence. As extensively shown in ovarian cancer, many missense mutations in *TP53*, specifically in the DNA-binding domain, not only result in loss of tumor suppression activity of the protein, but are also associated with protein overexpression and gain-of-function (GOF) properties that sustain the tumorigenic process [34, 48] and confer chemoresistance [49]. In colorectal cancer, *TP53* GOF mutations specifically activate the Wnt-β-catenin oncogenic signaling by increasing β-catenin protein levels through decreased degradation and enhanced synthesis [50]. Additionally, beyond acquiring a *TP53* mutation in one allele, most tumors lose the second allele by deletion or copy neutral LOH [51]. In keeping with these findings, increased β-catenin immunoreactivity was associated with p53 overexpression in ACC and, to an even greater extent, in the recurrence, whereas ACA showed a very low-level wild-type p53 staining pattern, supporting a GOF effect of the identified *TP53* mutation. LOH of *MEN1* may further contribute to dysregulation of Wnt-β-catenin oncogenic signaling, as menin has been shown to suppress cell proliferation through direct interaction with β-catenin [52]. Notably, in a large series of ovarian serous carcinomas, all *NF1-*mutated tumors also harbored a missense *TP53* mutation, suggesting a possible link between the two mutational events [53]. In ACC, the co-occurrence of *TP53* and *NF1* mutations has been reported to correlate with poorer overall and progression-free survival [17].

Although not recognized as ACC driver genes, the remaining 10 affected genes shared between ACA and ACC were also found to be mutated in the TCGA-ACC cohort, albeit at a low frequency. According to STRING analysis, 6 of them (*ATG12*,* GABRB3*,* IRF7*,* LCK*,* MAP4K1*, *SATB2*) are part of a predicted protein-protein interaction network centered in TP53, suggesting a potential role in the development of malignancy.

Clonal evolution analysis based on validated somatic variants identified four clonal populations in ACA, ACA + ACC, and ACC + recurrence. Clones 2 and 3, shared between ACA and ACC, corresponded to variants present in both lesions. Of note, clone 3 included two Tier III variants affecting *KANK1* and *REN.* Neither of these two clones was detected in the recurrence, consistently with the absence of ACA-ACC shared variants at this stage. Conversely, mutations in clones 1 and 4, which were shared between ACC and the recurrence, were not detected in ACA. Clone 1 included pathogenic variants in *TP53* and *NF1*, and its restriction to ACC and the recurrence suggests an association with the malignant phenotype and persistence during disease evolution. All selected genes affected by Tier III variants mapped to clone 1, indicating that this clone encompassed the variants with the highest predicted clinical relevance. Functional enrichment analysis of proteins encoded by genes mutated and shared between ACC and the recurrence revealed additional functional categories that were not observed among genes mutated in both ACA and ACC.

The absence of demonstrable biallelic *MEN1* inactivation in the adenoma is a major limitation of this study. Whole-exome and Sanger sequencing demonstrated LOH at the *MEN1* locus in ACC and the recurrence, while excluding classical coding alterations in the adenoma. However, the occurrence of alternative second-hit mechanisms, such as large genomic rearrangements, mutations in promoter or untranslated regions, or epigenetic alterations not detectable by WES, cannot be ruled out in ACA, particularly in the absence of informative menin immunohistochemistry. Consequently, a causal role for *MEN1* inactivation at the adenoma stage cannot be established.

An additional limitation is that this study is based on a single patient, which restricts the generalizability of the findings to the broader MEN1 patient population. Nonetheless, the spectrum of mutations identified is consistent with those reported in the TCGA-ACC dataset, supporting the biological relevance of these findings.

In conclusion, comprehensive molecular profiling of the ACA, ACC and recurrence occurring in a MEN1 patient has provided insight into the possible relationship between benign and malignant adrenocortical lesions in this context. Clonal evolution analysis identified distinct clonal populations across lesions, highlighting variants potentially relevant to malignant behavior and disease relapse. Although the clonal architecture and the number of variants shared between ACA and ACC are compatible with a clonal relationship between the two lesions, the lack of unequivocal evidence for *MEN1* inactivation in ACA precludes definitive conclusions regarding a stepwise adenoma-carcinoma progression versus independent multifocal tumor development within the same organ. Nonetheless, these findings support careful clinical consideration of the potential risk of ACC when managing MEN1 patients with adrenal lesions.

## Supplementary Information

Below is the link to the electronic supplementary material.

Supplementary Material 1 (DOCX 2.08 MB)

Supplementary Material 2 (DOCX 30.7 KB)

## Data Availability

The curated set of SNVs are reported as Supplementary File 2.xlsx
