# Supplementary material for: In-depth Genetic and Molecular Characterization of Unilateral Coexisting Adrenal Cortical Adenoma and Carcinoma in the Context of MEN1 Syndrome
Source: Endocr Pathol. 2026 Mar 10;37(1):14. doi: 10.1007/s12022-026-09908-0 (PMC12975836; doi:10.1007/s12022-026-09908-0)
Supplement: Supplementary file 1 — (DOCX 2.08 MB) [file 12022_2026_9908_MOESM1_ESM.docx]

**SUPPLEMENTARY FILE**

**Materials & Methods**

*Pathological analysis*

Tumor specimens were evaluated according to the Weiss scoring system [6,15]. The mitotic count was assessed in the most mitotically active part of the tumor and recorded per 10 mm^2^. Tumor stage was assessed according to the TNM classification of ACC proposed by ENSAT [6]. Completeness of surgery was established by resection status: R0, free resected margins; R1, microscopic involvement of resected margins; R2, macroscopic invasion of resected margins; RX, not determined.

The reticulin stain was performed using a commercially available silver impregnation kit on an automated Benchmark Special Stains system (Ventana Medical System, Tucson, AZ, USA).

Formalin-fixed paraffin-embedded (FFPE) tissue sections were stained using a BenchMark XT automated immunostaining system (Ventana Medical System, Tucson, AZ, USA). Details on primary antibodies used for immunohistochemical analysis are listed in **Tab.S1.**

The Ki67 proliferative index was measured by manual counting in areas showing the highest proliferative activity. Ki67-positive nuclei were counted in 1000 tumor cells, and the index was calculated by dividing the number of positive cells (any cell with nuclear staining was counted as positive) by the total number of malignant cells.

*Transcriptome signature*

RNA was extracted from formalin-fixed paraffin-embedded (FFPE) tumor samples using RNeasy DSP FFPE Kit (Qiagen) following the manufacturer's protocol. Sequencing libraries were prepared at the Genomics Platform of Institut Cochin, following the QuantSeq 3’ mRNA-Seq protocol (Lexogen). Single read sequencing (1x75bp) was performed on a NextSeq 550 platform (Illumina). FASTQ sequences were aligned on the GRCh38 human reference genome with STAR (v.2.7.10b). Read counts were normalized on sequencing depth (counts per million reads) then log-transformed. A ridge penalized regression model, based on the 2000 most variable genes in the reference cohort, was used to classify study samples into “C1A” (poor outcome ACC), “C1B” (better outcome ACC) or “C2” (ACA), as previously described [16]. Study samples were then projected on the PCA of the reference cohort for visualization.

**Tier classification**

We classified the prioritized somatic variants, according to the joint consensus recommendation of the Association for Molecular Pathology, American Society of Clinical Oncology, and College of American Pathologists [28]. This classification is based on the clinical impact of each variant and consists of four categories:

- **Tier I**, i.e. variants of strong clinical significance based on level A and B evidence.

Ø Level A evidence: FDA-approved therapy included in professional guidelines

Ø Level B evidence: well-powered studies with consensus from experts in the field

- **Tier II**, variants of potential clinical significance based on level C or D evidence.

Ø Level C evidence: FDA-approved therapy for different tumor types or investigational therapies

Ø Level D: preclinical trials or a few case reports without consensus

- **Tier III**, variants of unknown clinical significance. These variants are not observed at a significant allele frequency in the general or specific subpopulation databases, or pan-cancer or tumor-specific variant databases. In addition, no convincing published evidence of cancer association is reported.

- **Tier IV**, variants that are benign or likely benign. These variants are observed at significant allele frequency in the general or specific subpopulation databases. There is no published evidence of cancer association.

**Supplementary Table**

| **Antibody (clone)** | **Source** | **Dilution** |
| --- | --- | --- |
| AE1/AE3 (PCK26) | Roche | Pre-diluted |
| S100 Protein (4C4.9) | Roche | Pre-diluted |
| SF-1 (EPR19744) | Abcam | 1:500 |
| Chromogranin-A (LK2H10) | Roche | Pre-diluted |
| Synaptophysin (SP11) | Roche | Pre-diluted |
| MART-1/Melan-A (A103) | Roche | Pre-diluted |
| p53 (DO-7) | Roche | Pre-diluted |
| Beta-catenin (14) | Cell Marque | Pre-diluted |
| Ki-67 (30-9) | Roche | Pre-diluted |
| IGF2 | Abcam | 1:100 |

**Tab S1. Primary antibodies for immunohistochemical analysis**

**Supplementary Figures**


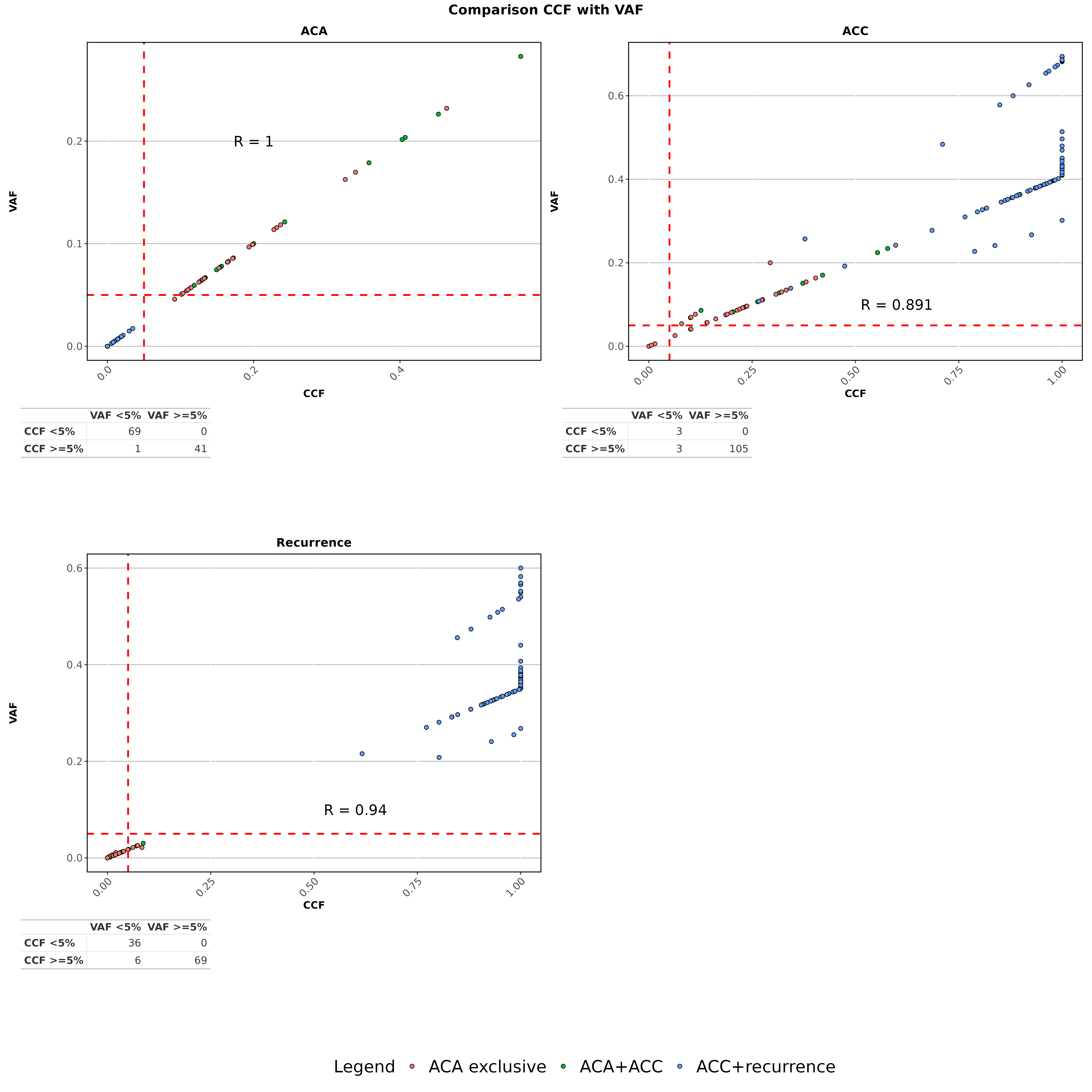


**Fig. S1** Scatter plot showing VAF (y-axis) versus CCF (x-axis) in ACA, ACC and relapse samples. The contingency table reports number of variants classified based on 5% threshold by VAF or CCF.


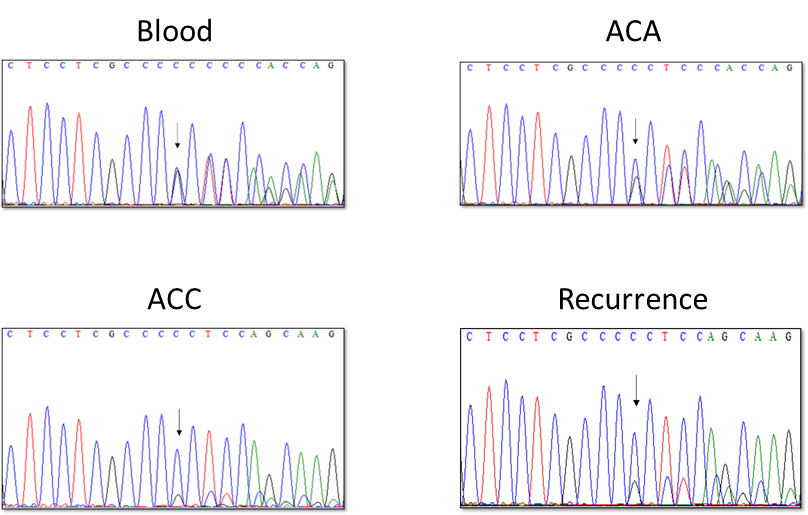


**Fig. S2** Sequence electropherograms of exon 8 of the *MEN1* gene in different tissues, showing the presence of the frameshift mutation that consists of a cytosine deletion in ACC and recurrence, not present in ACA and in germline DNA, at position 1154 of cDNA, indicated by the arrow.


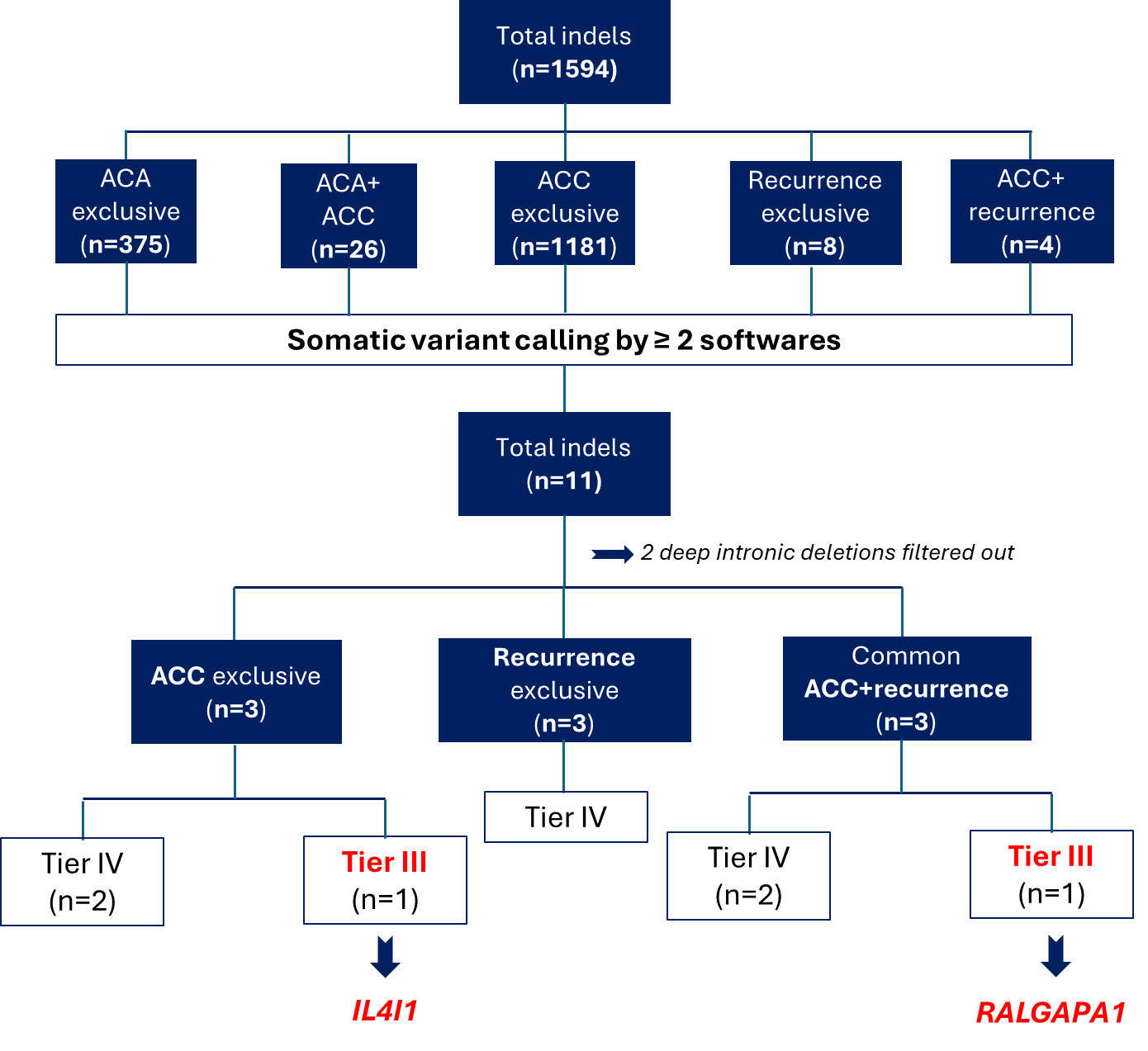


**Fig. S3** Flowchart illustrates the indels filtering approach based on variant calling by at least 2 out of the 3 software used (Mutect2-GATK, Strelka and VarScan). Red color indicates affected genes by variants of unknown significance (Tier III). Tier IV: variants classified as likely benign/benign; Tier III: variants of unknown clinical significance

**
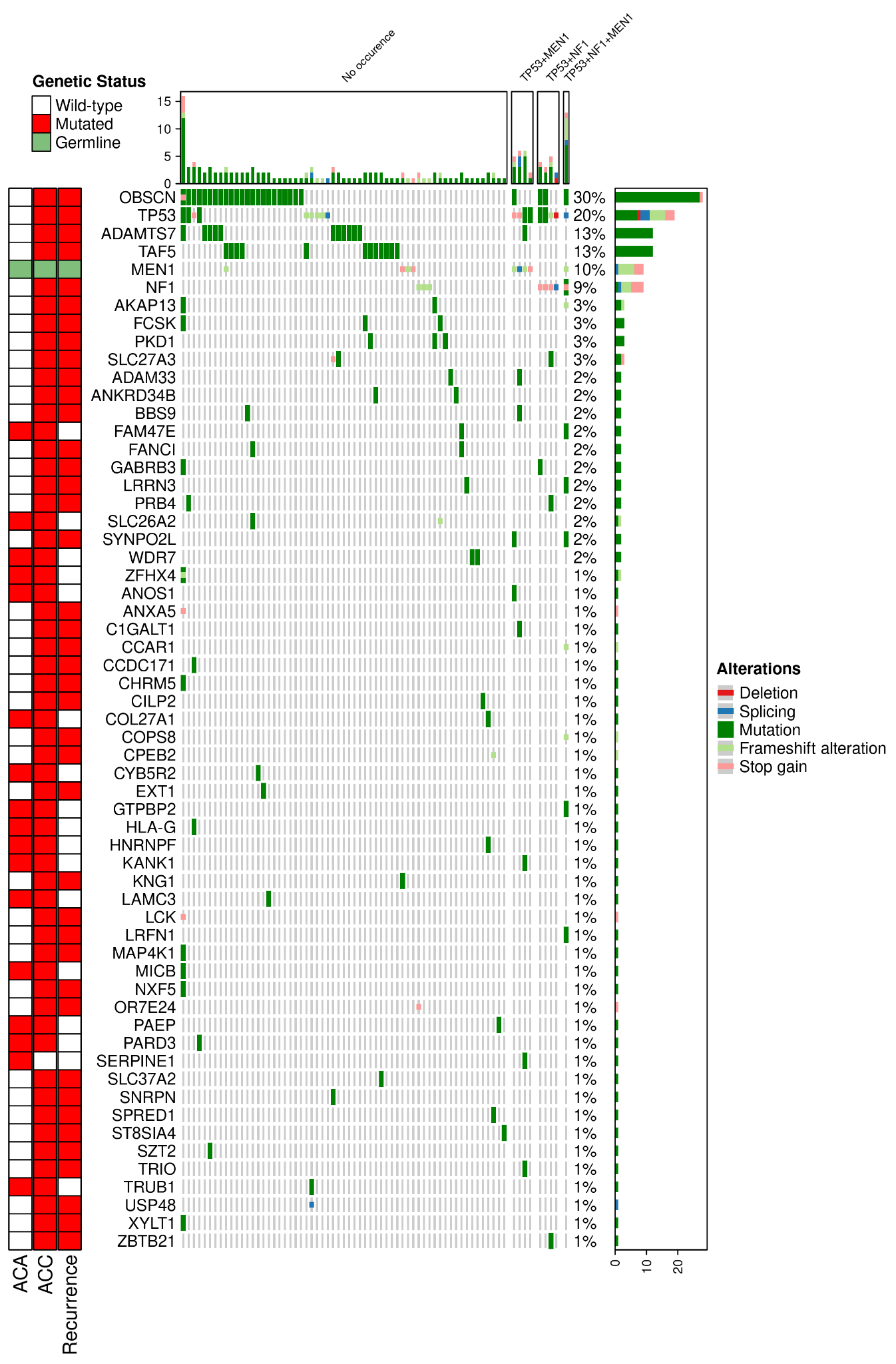
**

**Fig. S4 Heat map comparing mutated genes found by WES in ACA, ACC and recurrence tumor samples compared with data obtained in the TCGA-ACC database.** Mutational data of TCGA-ACC Firehose Legacy were retrieved from cBioPortal (The Cancer Genome Atlas Research Network, 2016) and mutated genes of ACA, ACC and recurrence, along with *MEN1*, were queried (n=106, TCGA-ACC sample n = 90). The OncoPlot shows only the genes that are mutated in at least one TCGA sample (n=59) and only those samples that have at least one mutation queried (n=70). Genes are sorted from the most mutated from the least mutated. The mutation type is reported in the right legend. Top annotation is the number of mutations for each sample and right annotation is the percentage of samples mutated for that gene (in %). Left annotation keeps track of mutation occurrence of that gene in the three conditions: red if mutated, white if not and green if the mutation is germline (only MEN1). Samples are split in different columns if the following conditions were matched: TP53+MEN1 mutated (n=4), TP53+NF1 mutated (n=4) and TP53+NF1+MEN1 mutated (n=1, TCGA id = TCGA-PK-A5HB-01). The split highlights those samples that are genetically similar to tumoral conditions of our case, regarding somatic mutational status


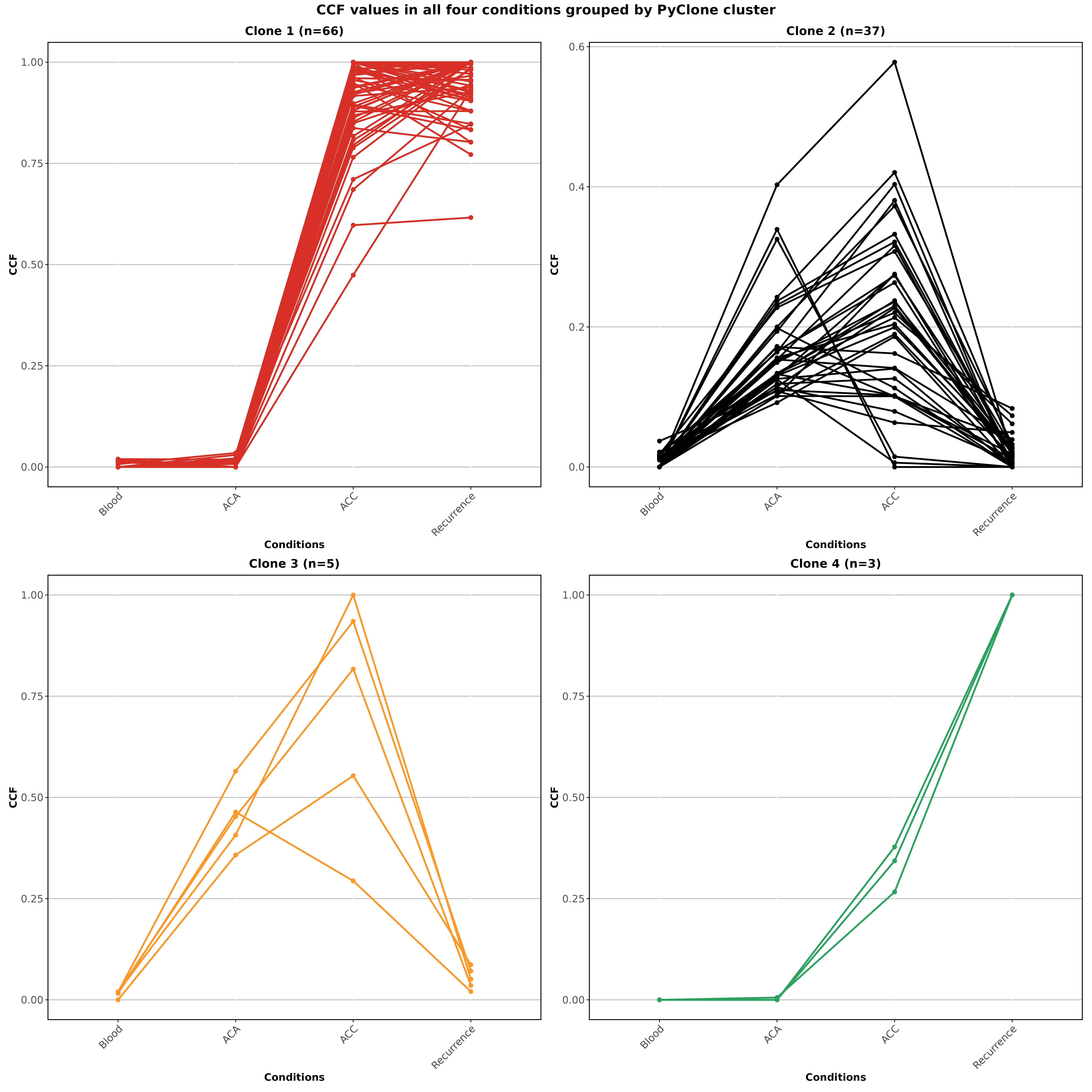


**Fig. S5** Spaghetti plots showing CCF across the different sample types across the four clusters identified by PyClone.
