# Supplementary material for: In-depth Genetic and Molecular Characterization of Unilateral Coexisting Adrenal Cortical Adenoma and Carcinoma in the Context of MEN1 Syndrome
Source: Endocr Pathol. 2026 Mar 10;37(1):14. doi: 10.1007/s12022-026-09908-0 (PMC12975836; doi:10.1007/s12022-026-09908-0)
Supplement: Supplementary file 2 — (DOCX 30.7 KB) [file 12022_2026_9908_MOESM2_ESM.docx]

| Cluster | Chromosome | Position | Gene | Ref | Alt | ACA | ACC | Recurrence |
| --- | --- | --- | --- | --- | --- | --- | --- | --- |
| 1 | 1 | 21706833 | USP48 | C | T | - | + | + |
| 1 | 1 | 32275415 | LCK | G | A | - | + | + |
| 1 | 1 | 43425593 | SZT2 | G | T | - | + | + |
| 2 | 1 | 152313789 | FLG-AS1 | C | G | + | + | - |
| 1 | 1 | 153779159 | SLC27A3 | C | T | - | + | + |
| 2 | 1 | 204155884 | REN | G | T | + | + | - |
| 2 | 1 | 204155886 | REN | G | A | + | + | - |
| 1 | 1 | 228305315 | OBSCN | C | T | - | + | + |
| 1 | 2 | 47162624 | CALM2 | C | A | - | + | + |
| 1 | 2 | 177552445 | IFT70B | C | G | - | + | + |
| 1 | 2 | 199308953 | SATB2 | C | T | - | + | + |
| 1 | 2 | 237094179 | COPS8 | G | A | - | + | + |
| 4 | 3 | 186742260 | KNG1 | G | T | - | + | + |
| 4 | 4 | 15003932 | CPEB2 | C | T | - | + | + |
| 1 | 4 | 17876755 | LCORL | C | T | - | + | + |
| 2 | 4 | 39504478 | UGDH | G | A | + | - | - |
| 2 | 4 | 76278257 | FAM47E | G | A | + | + | - |
| 2 | 4 | 76278259 | FAM47E | T | C | + | + | - |
| 1 | 4 | 121683443 | ANXA5 | C | T | - | + | + |
| 1 | 4 | 149553151 | IQCM | C | A | - | + | + |
| 1 | 5 | 14368886 | TRIO | A | G | - | + | + |
| 1 | 5 | 80559297 | ANKRD34B | C | A | - | + | + |
| 1 | 5 | 100811938 | ST8SIA4 | G | A | - | + | + |
| 1 | 5 | 115831811 | ATG12 | C | T | - | + | + |
| 2 | 5 | 135852678 | SLC25A48 | G | T | + | + | - |
| 3 | 5 | 149981866 | SLC26A2 | C | G | + | + | - |
| 3 | 5 | 149981870 | SLC26A2 | C | A | + | + | - |
| 2 | 5 | 176368950 | ARL10 | G | C | + | - | - |
| 2 | 6 | 324973 | DUSP22 | C | G | + | + | - |
| 1 | 6 | 26104160 | H4C3 | C | T | - | + | + |
| 3 | 6 | 29829672 | HLA-G | G | C | + | + | - |
| 2 | 6 | 31507311 | MICB | G | C | + | + | - |
| 2 | 6 | 31507312 | MICB | G | C | + | + | - |
| 2 | 6 | 43621603 | GTPBP2 | G | A | + | + | - |
| 1 | 7 | 7182811 | C1GALT1 | C | G | - | + | + |
| 1 | 7 | 33505512 | BBS9 | A | G | - | + | + |
| 1 | 7 | 66087376 | ASL | G | A | - | + | + |
| 1 | 7 | 87196699 | TMEM243 | G | C | - | + | + |
| 1 | 7 | 95592057 | PDK4 | C | G | - | + | + |
| 1 | 7 | 97736343 | TAC1 | T | G | - | + | + |
| 1 | 7 | 98107265 | LMTK2 | C | A | - | + | + |
| 2 | 7 | 101133872 | SERPINE1 | C | T | + | - | - |
| 1 | 7 | 111123068 | LRRN3 | T | C | - | + | + |
| 1 | 8 | 39935195 | IDO2 | G | C | - | + | + |
| 2 | 8 | 76704571 | ZFHX4 | T | C | + | + | - |
| 1 | 8 | 117837133 | EXT1 | G | C | - | + | + |
| 2 | 8 | 125431142 | TRIB1 | A | G | + | + | - |
| 2 | 9 | 738293 | KANK1 | T | A | + | + | - |
| 1 | 9 | 15744619 | CCDC171 | G | A | - | + | + |
| 1 | 9 | 34483460 | DNAI1 | G | C | - | + | + |
| 2 | 9 | 114290248 | COL27A1 | C | T | + | + | - |
| 2 | 9 | 131096670 | LAMC3 | G | C | + | + | - |
| 2 | 9 | 135565346 | PAEP | T | C | + | + | - |
| 1 | 10 | 6221461 | PFKFB3 | C | T | - | + | + |
| 2 | 10 | 34111190 | PARD3 | T | C | + | + | - |
| 3 | 10 | 43386718 | HNRNPF | G | T | + | + | - |
| 1 | 10 | 68788149 | CCAR1 | G | C | - | + | + |
| 1 | 10 | 71851321 | PSAP | A | G | - | + | + |
| 1 | 10 | 73654151 | SYNPO2L | G | A | - | + | + |
| 2 | 10 | 75029928 | KAT6B | A | C | + | + | - |
| 1 | 10 | 86942853 | MMRN2 | G | A | - | + | + |
| 1 | 10 | 99955436 | DNMBP | G | A | - | + | + |
| 1 | 10 | 103383264 | TAF5 | G | C | - | + | + |
| 2 | 10 | 114938346 | TRUB1 | G | T | + | + | - |
| 1 | 11 | 613103 | IRF7 | G | A | - | + | + |
| 2 | 11 | 7672873 | CYB5R2 | T | C | + | + | - |
| 1 | 11 | 125086230 | SLC37A2 | G | A | - | + | + |
| 1 | 11 | 126005894 | CDON | G | A | - | + | + |
| 1 | 12 | 11308800 | PRB4 | G | T | - | + | + |
| 1 | 12 | 124912641 | UBC | G | A | - | + | + |
| 2 | 14 | 22770610 | OXA1L | G | C | + | + | - |
| 1 | 15 | 24829895 | SNRPN | C | A | - | + | + |
| 1 | 15 | 26883469 | GABRB3 | A | G | - | + | + |
| 1 | 15 | 34062864 | CHRM5 | C | G | - | + | + |
| 1 | 15 | 38352241 | SPRED1 | G | C | - | + | + |
| 2 | 15 | 62067803 | C2CD4A | C | G | + | - | - |
| 1 | 15 | 64162082 | PPIB | G | A | - | + | + |
| 1 | 15 | 78766243 | ADAMTS7 | T | C | - | + | + |
| 1 | 15 | 85727203 | AKAP13 | A | G | - | + | + |
| 1 | 15 | 88531192 | DET1 | C | T | - | + | + |
| 1 | 15 | 89318023 | FANCI | G | A | - | + | + |
| 2 | 16 | 1351812 | TSR3 | A | C | + | + | - |
| 2 | 16 | 1351814 | TSR3 | C | G | + | + | - |
| 1 | 16 | 1773326 | EME2 | C | G | - | + | + |
| 1 | 16 | 2114627 | PKD1 | C | T | - | + | + |
| 2 | 16 | 4894622 | PPL | G | C | + | + | - |
| 2 | 16 | 13948604 | ERCC4 | C | T | + | + | - |
| 1 | 16 | 17127684 | XYLT1 | C | T | - | + | + |
| 4 | 16 | 70473037 | FCSK | C | T | - | + | + |
| 1 | 17 | 7675236 | TP53 | A | C | - | + | + |
| 1 | 17 | 31259031 | NF1 | G | C | - | + | + |
| 2 | 18 | 56816072 | WDR7 | C | T | + | + | - |
| 2 | 19 | 1229521 | CBARP | G | T | + | - | - |
| 2 | 19 | 1229758 | CBARP | T | C | + | - | - |
| 1 | 19 | 9251463 | OR7E24 | G | C | - | + | + |
| 2 | 19 | 12897383 | GCDH | G | T | + | + | - |
| 1 | 19 | 19545796 | CILP2 | C | T | - | + | + |
| 1 | 19 | 38609971 | MAP4K1 | C | G | - | + | + |
| 1 | 19 | 39314061 | LRFN1 | G | A | - | + | + |
| 1 | 19 | 58547416 | TRIM28 | C | T | - | + | + |
| 1 | 19 | 58556217 | UBE2M | C | A | - | + | + |
| 1 | 20 | 3674850 | ADAM33 | C | A | - | + | + |
| 1 | 21 | 41992898 | ZBTB21 | G | C | - | + | + |
| 1 | 22 | 22906657 | IGLC3 | C | G | - | + | + |
| 3 | 22 | 23394409 | ZDHHC8P1 | G | T | + | + | - |
| 2 | X | 8585395 | ANOS1 | G | C | + | + | - |
| 1 | X | 12791540 | PRPS2 | C | G | - | + | + |
| 2 | X | 51408161 | EZHIP | A | T | + | + | - |
| 2 | X | 51618674 | CENPVL3 | G | A | + | + | - |
| 2 | X | 54922610 | TRO | T | C | + | + | - |
| 1 | X | 101841707 | NXF5 | G | T | - | + | + |
